# Mesenteric lymphatic malformation associated with acute appendicitis: a case report

**DOI:** 10.4076/1752-1947-3-9030

**Published:** 2009-09-17

**Authors:** Catherine Hunter, Meghan Connelly, Steven Lee, Larry Wang, Nam Nguyen

**Affiliations:** 1 Department of Surgery, Keck School of Medicine, University of Southern California, Los Angeles, California 90089, USA; 2 Department of Surgery, Harbor UCLA Medical Center, W Carson Street, Torrance, California 90502, USA; 3 Childrens Hospital Los Angeles, Sunset Blvd, Los Angeles, California 90027, USA; 4 Department of Surgery, Kaiser Permanente, 4700 Sunset Blvd, Los Angeles, California 90027, USA; 5 Department of Pathology, Keck School of Medicine, University of Southern California, Los Angeles, California 90089, USA

## Abstract

**Introduction:**

Mesenteric lymphatic malformations are rare, benign tumors that are most commonly found in children. The presentation of these tumors is variable and may either be innocuous or life threatening. It has been suggested that mesenteric lymphatic malformations are congenital; however, there is evidence that their growth may be stimulated by local trauma.

**Case presentation:**

We describe the first case of a mesenteric lymphatic malformation associated with acute appendicitis in a 13-year-old Caucasian boy. The patient is well six months after surgical excision of the tumor.

**Conclusion:**

The reader should be aware that growth and/or development of mesenteric lymphatic malformations may be associated with trauma and other pro-inflammatory processes.

## Introduction

Mesenteric lymphatic malformations (MLM) are rare, benign tumors that most commonly develop in children [1]. The nomenclature of lymphatic malformations is at times confusing; microcystic lymphatic malformation was previously called lymphangioma and macrocystic was called cystic hygroma. Lymphangiomas are commonly located in the skin and subcutaneous tissues, although they have been described in deeper tissues including the neck, axilla, and retroperitoneum. The incidence of intra-abdominal MLM is low, with less than 200 cases in the literature [2]. One institution reported 193 cases of children with lymphangiomas, with the following distribution: cervical (31.4%), craniofacial (18.9%), extremity (18.9%), trunk (9.2%), abdominal (9.2%), cervicoaxillothoracic (4.9%), cervicomediastinal (2.2%), intrathoracic (1.6%) and multiple (3.8%) [3]. The etiology of lymphatic malformations is unclear. They may be congenital or may develop secondary to infection or trauma. We present the first case report of a child who developed MLM associated with acute appendicitis.

## Case presentation

A 13-year-old Caucasian boy presented with acute appendicitis, characterized by right lower quadrant pain. He underwent a laparoscopic appendectomy, and a pathology report confirmed the diagnosis of focal acute appendicitis. No intra-abdominal masses were noted at the time of surgery. However, after the operation, the patient experienced persistent drainage of serosanginous fluid from a trochar site. Once this drainage ceased, the patient was discharged home ten days after admission.

**Figure 1:**
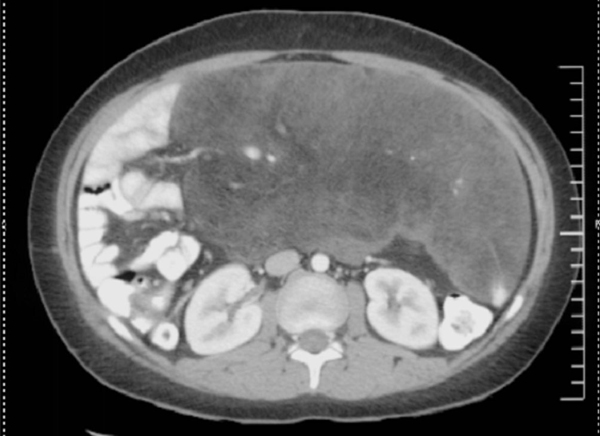
**A computed tomography of a large intra-abdominal mass is seen with displacement of the surrounding structures**.

During the next six months he continued to experience intermittent abdominal pain, which led to representation to the emergency room. Additionally, the patient experienced an increase in abdominal girth, abdominal pain, and weight loss. No constipation, diarrhea, nausea, vomiting or jaundice was reported. A CT scan of the abdomen and pelvis demonstrated a 23 by 12.5 cm fatty soft tissue mass surrounded by mesenteric fat (Figure 1). The tumor encased branches of the superior mesenteric artery and displaced the small bowel. Multiple fluid collections were also seen in the right lower quadrant. A subsequent CT-guided biopsy suggested a benign fatty tumor; the differential diagnosis included lymphangioma, lipoma and fibrolipoma. The patient was then transferred to our institution for definitive care.

A physical examination of the patient revealed that his vital signs were within normal limits and that his abdomen was distended with a large palpable mass extending from the left flank to the right semilunar line. A large part of the bowel appeared to be displaced in the right abdomen, and the presence of shifting dullness suggested ascites.

**Figure 2:**
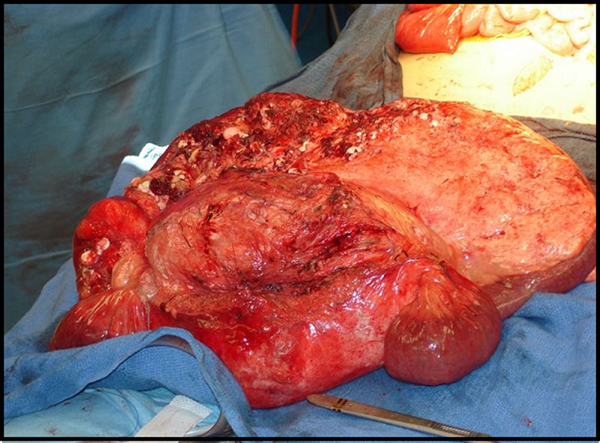
**An intra-operative image of the huge circumscribed mass is located in a small bowel serosal region with milky secretion on the surface**.

**Figure 3:**
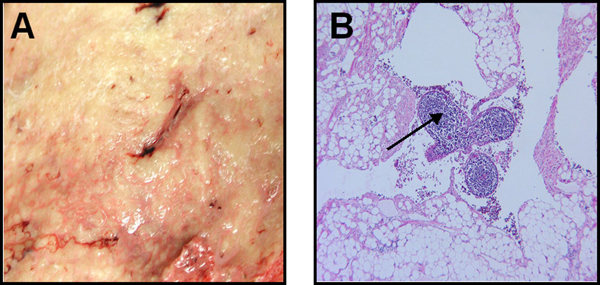
**Pathologic imaging of the mesenteric lymphatic malformations**. **Panel A**: The tumor is not encapsulated and has a spongy appearance with multiple pinhead-sized spots in the peripheral areas. The dilated lymphatic vessels are filled with a milk-white fluid. **Panel B**: (×200) The abnormally dilated endothelial-lined spaces contain lymphocytic aggregates (arrow).

The patient was taken to the operating room where a diagnostic laparoscopy confirmed a large intra-abdominal tumor arising from the mesentery and a significant amount of free chylous fluid. The tumor adhered to the duodenum and the superior mesenteric artery (SMA) and vein (SMV). A frozen biopsy performed during the operation suggested that the mass was a lymphatic vascular malformation with a fibrous stroma and fibrous capsule consistent with a benign tumor. The operation was converted to a midline laparotomy for tumor resection. As mentioned earlier, the tumor was largely entangled with the mesentery. However, a circumferential dissection was performed through the creation of a plane between the tumor and mesentery. The SMA, the third and fourth portions of the duodenum, and the proximal small bowel were each affixed to the tumor but were successfully dissected off. The tumor was excised along with 50 cm of small bowel, and a primary anastamosis was performed (Figure 2). The final pathology demonstrated a 27.5-cm lymphatic malformation with no evidence of malignancy (Figure 3); the ascitic fluid aspiration was deemed to be chylous ascites. The patient recovered well and was discharged home eight days after the operation. At a follow-up visit six months after the operation, the patient is doing well and tolerating full oral feeding, with complete resolution of his abdominal complaints.

## Discussion

MLM are rare with reported frequencies of 1 in 20,000 to 1 in 250,000 admissions [2]. However, the precise number of MLM cases is unclear because most reports do not clearly differentiate between MLM and other mesenteric cysts. Although MLM may occur at any age, most cases of MLM are found in children, with an increasing number of cases diagnosed before birth [4]. In fact, almost 60% of cases present prior to the fifth year of life. MLM have been described in both men and women; however, some studies have demonstrated a male predominance [5]. The presentation of MLM is variable, and the clinical symptoms may include pain, nausea or vomiting [5]. Mesenteric lymphatic malformations may be misdiagnosed as benign abdominal processes or even as malignancies [6]. Although most cases of MLM are discovered by accident, they may be found in association with intra-abdominal catastrophes such as intestinal volvulus [7]. Additionally, traumatic hemorrhage in MLM has been described [8]; however, there are no reports of MLM developing as a result of intra-abdominal trauma, surgery or infection.

The etiology of MLM is unknown, although it has been proposed that they may be associated with developmental anomalies of the lymphatic system, secondary to a failure of the lymphatic system to connect with the venous system. This theory may, in part, be supported by the predominance of MLM in children. Other possible causes include inflammation in the lymphatic channels, resulting in obstruction and subsequent lymphangioma formation [9]. It has further been suggested that injury may trigger delayed proliferation of cells, thereby causing lymphatic malformations to develop [10]. Although there have been reports of injury and infection associated with lymphatic malformations in the extremities and neck [11]-[13], there is only one report of a lymphatic malformation developing after surgery, and this is the case of a retroperitoneal cyst which developed after cholecystectomy [14].

A highly unusual feature of this case report is that the patient's MLM apparently developed in connection with his appendicitis. This raises one of two possibilities. First, it is possible that the patient's mass was simply not seen during the first surgery. This is possible since persistent drainage from the trochar sites complicated the patient's condition after the operation, suggesting that the MLM may have already been present. The second possibility is that the appendicitis or the surgery and appendectomy triggered an exponential growth of the MLM. Since no imaging was obtained during the initial diagnosis, there is no way to determine conclusively whether or not the mass was absent. However, it certainly appears that the mass developed, or more probably, grew exponentially after the appendectomy.

## Conclusions

MLM are rare tumors that are more common among children. They may be associated with a range of clinical symptoms and are best treated with surgical resection since they may grow to a large size, compress vital structures and cause intra-abdominal catastrophe. We describe the first case of development of a MLM associated with appendicitis and suggest that the trauma of surgery may have triggered the exponential growth and development of this tumor. We recommend that surgeons and pediatric health care providers be aware of this association and consider it in their differential diagnoses.

## Abbreviations

CT: computed tomography; MLM: mesenteric lymphatic malformations; SMA: superior mesenteric artery; SMV: superior mesenteric vein.

## Competing interests

The authors declare that they have no competing interests.

## Consent

Written informed consent was obtained from the patient's parents for publication of this case report and any accompanying images. A copy of the written consent is available for review by the Editor-in-Chief of this journal.

## Authors' contributions

CH obtained the images and wrote the manuscript. LW prepared the images. MC, SL, and NN contributed significantly to the writing of this manuscript.

## References

[B1] Konen O Rathaus V Dlugy E Freud E Kessler A Shapiro M Horev G Childhood abdominal cystic lymphangioma Pediatr Radiol 2002 32 88 94 10.1007/s00247-001-0612-4 11819071

[B2] Losanoff JE Richman BW El-Sherif A Rider KD Jones JW Mesenteric cystic lymphangioma J Am Coll Surg 2003 196 598 603 10.1016/S1072-7515(02)01755-6 12691938

[B3] Hancock BJ St-Vil D Luks FI Di Lorenzo M Blanchard H Complications of lymphangiomas in children J Pediatr Surg 1992 27 220 224 10.1016/0022-3468(92)90316-Y 1564622

[B4] Merrot T Chaumoitre K Simeoni-Alias J Alessandrini P Guys JM Panuel M Abdominal cystic lymphangiomas in children. Clinical, diagnostic and therapeutic aspects: apropos of 21 cases Ann Chir 1999 53 494 499 10427841

[B5] Takiff H Calabria R Yin L Stabile BE Mesenteric cysts and intra-abdominal cystic lymphangiomas Arch Surg 1985 120 1266 1269 4051731 10.1001/archsurg.1985.01390350048010

[B6] Kirzeder DJ Kan JH Mesenteric lymphatic malformation Pediatr Radiol 2007 37 845 846 10.1007/s00247-007-0521-2 17546452

[B7] Traubici J Daneman A Wales P Gibbs D Fecteau A Kim P Mesenteric lymphatic malformation associated with small-bowel volvulus - two cases and a review of the literature Pediatr Radiol 2002 32 362 365 10.1007/s00247-002-0658-y 11956726

[B8] Porras-Ramirez G Hernandez-Herrera MH Hemorrhage into mesenteric cyst following trauma as a cause of acute abdomen J Pediatr Surg 1991 26 847 848 10.1016/0022-3468(91)90153-K 1895196

[B9] Gleason TJ Yuh WT Tali ET Harris KG Mueller DP Traumatic cervical cystic lymphangioma in an adult Ann Otol Rhinol Laryngol 1993 102 564 566 8333679 10.1177/000348949310200714

[B10] Wiggs WJ Jr Sismanis A Cystic hygroma in the adult: two case reports Otolaryngol Head Neck Surg 1994 10 239 241 10.1177/019459989411000216 8108161

[B11] Postacchini F Sadun R Lymphangioma of the thigh following acute trauma Clin Orthop Relat Res 1976 121 169 172 991499

[B12] Antoniades K Kiziridou A Psimopoulou M Traumatic cervical cystic hygroma Int J Oral Maxillofac Surg 2000 29 47 48 10.1016/S0901-5027(00)80124-1 10691144

[B13] Aneeshkumar MK Kale S Kabbani M David VC Cystic lymphangioma in adults: can trauma be the trigger? Eur Arch Otorhinolaryngol 2005 262 335 337 10.1007/s00405-004-0780-6 15841412

[B14] Niwa H Sumita N Ishihara K Hoshino T Iwase H Kuwabara Y A case of retroperitoneal chylous cyst developed after cholecystectomy and choledochotomy Nippon Geka Gakkai Zasshi 1988 89 282 285 3362131

